# Sleep

**Published:** 1856-12

**Authors:** 


### SLEEP.

The unwisest of all economies is time saved from necessary sleep, for it begets a nervous irritability, which masters the body and destroys the mind. When a man becomes sleepless, the intellect is in danger. A restored lunatic, of superior mental endowments, said: "The first symptom of insanity, in my own case, was a want of sleep; and from the time I began to sleep soundly, my recovery was sure."

Let this be a warning to all who are acquiring an education. Every young person at school should have eight hours for sleep out of every twenty-four; for, as the brain is highly stimulated all the time, in the prosecution of study, it will break down, just as any other part of the frame, unless it have time for full recuperation. Better, a thousand times, to give another year to the completion of specified studies, than by curtailing sleep, to endeavor to get through that much sooner, at the risk of madness.

